# The effect of mental health interventions on psychological distress for informal caregivers of people with mental illness: A systematic review and meta-analysis

**DOI:** 10.3389/fpsyt.2022.949066

**Published:** 2022-10-06

**Authors:** Nanja Holland Hansen, Lasse Bjerrekær, Karen Johanne Pallesen, Lise Juul, Lone Overby Fjorback

**Affiliations:** Department of Clinical Medicine, Danish Center for Mindfulness, University of Aarhus, Aarhus, Denmark

**Keywords:** interventions, informal caregivers, mental health, psychological distress, systematic review, meta-analysis

## Abstract

**Introduction:**

Informal caregivers of people with a mental illness are at increased risk of developing depression, anxiety, and stress, so preventive interventions are needed.

**Method:**

The review was reported in PROSPERO (ID: CRD42018094454). The PsycINFO, PubMed, and Scopus databases were searched in June 2019. The Cochrane Risk of Bias and Jadad scale scores were used to assess study quality. Inclusion criteria were: RCTs of informal caregiver interventions regardless of the care receiver's mental illness and intervention modality. Interventions should be compared to a waitlist, treatment as usual or active control, taught in real-time by a mental health professional, include an outcome measure on psychological distress, and published in a peer-reviewed journal article in English. RCTs were excluded if the intervention was given in dyads (caregiver + care receiver), limited to the provision of respite care where the patient sample included a mix of both physical and psychological illnesses, unpublished, not peer-reviewed, study protocols, or dissertations.

**Results:**

A total of 2,148 studies were identified; of these, 44 RCT studies met the inclusion criteria, and 31 had sufficient data to conduct a meta-analysis including subgroup analysis (*N* = 1,899). The systematic review showed that thirty-one out of the 44 RCTs had an effect of the intervention on decreasing psychological distress. The results of the meta-analysis, which included informal caregiver interventions, compared to waitlist, treatment as usual, or active control, regardless of care-receiver mental illness or intervention modality showed a small effect of −0.32 (95% CI −0.53 to −0.11). The heterogeneity of the included studies was high (*I*^2^ = 78). The subgroup analysis included manualized interventions lasting at least 8 weeks and the subgroup analysis that included an active control showed a small effect and low heterogeneity. Lack of active control and long-term follow-up is a limitation of most of the studies.

**Conclusion:**

The evidence supports that several interventions improve the mental health of caregivers. Manualized interventions ≥ 8 weeks with active participation are most effective. Future RCTs should improve methodology, and research should investigate which intervention modality is most effective for what kind of caregiver. Future research should clearly specify what the included intervention components are, use longer follow-up times, and conduct mediational analyses to better understand what mechanisms create the effect of an intervention.

**Systematic review registration:**

Identifier: CRD42018094454.

## Introduction

Informal caregivers of people with a mental illness are at raised risk of mental health difficulties such as depression, stress, and anxiety (1–5). In the United Kingdom alone, it is estimated that 9 million people will provide informal care for a loved one by 2037 (5), and in Denmark, 61% of informal caregivers state that they experience psychological distress because of their care-giving role (6). Direct expression of the needs of caregivers has been neglected or received little attention, and a key obstacle to the provision of relevant support to informal caregivers is that they are often not identified (7). Government agencies such as the National Institute for Health and Care Excellence (7) recommend providing informal caregivers with interventions that help them with skills needed to take care of their own mental health (8).

There is a call for evidence-based interventions for caregivers (9). Individual studies have investigated intervention effects on informal caregiver distress regardless of care-receiver mental illness (10), with results suggesting that being an informal caregiver regardless of care-receiver mental illness causes symptoms of psychological distress (5, 11). Systematic and meta-analytic reviews of intervention programs for informal caregivers have found conflicting evidence for the effectiveness of intervention programs on psychological distress (12). The literature tends to categorize interventions based on the type of mental illness. Broadly, there are two categories, with both categories being considered interventions for informal caregivers of people suffering from a mental illness: (1) caregiver interventions for people with dementia/Alzheimer's disease and (2) caregiver interventions for people with a severe mental illness (usually including psychosis, schizophrenia, and mood disorders).

Regarding the first broad category, systematic reviews and meta-analyses found evidence that individual behavioral management therapy focusing on care-recipient behavior is helpful in alleviating informal caregiver symptoms of psychological distress (13). A comprehensive meta-review found that many different types of interventions (i.e., multicomponent, mindfulness, psychoeducation, and occupational therapy) were helpful in targeting symptoms of depression, quality of life, mastery, and communication with little effect of interventions on anxiety (14). Moreover, systematic reviews and meta-analyses have found conflicting results regarding intervention format (14, 15), with some favoring individual format (8) and others group format (13).

Duration and components (such as structure) that are needed for an intervention to show effect are also a debated issue within the literature, with systematic reviews suggesting 7–9 sessions (8) and others suggesting at least 6 sessions (13). Moreover, the results of a meta-analysis suggested that structured and more intensive interventions (16) with active participation show a greater effect than less structured interventions (17). On the other hand, two recently published studies showed that shorter interventions of 4 or 5 sessions showed the same effect as longer (8 sessions) interventions (18, 19). Taken together, the conflicting evidence regarding duration and components such as structure remains an issue in the literature.

The limitations found in systematic reviews and meta-analyses included a lack of methodological rigor and detailed descriptions of the interventions to make them easier to classify and understand which components in different interventions are driving the effect (8, 13–15, 17).

The second broad group of caregiver intervention studies was for informal caregivers of people with severe mental illnesses (psychosis, schizophrenia spectrum, and mood disorder). Two systematic reviews and one meta-analysis investigated the effects of caregiver interventions on the decreased burden and emotional response, and intervention components such as; duration, delivery mode, structure, and content (12, 20, 21). The results showed that psychoeducation decreased psychological distress and subjective burden (12, 21), and that support groups and bibliotherapy were also effective in decreasing psychological distress (12). No evidence was found to suggest that the presence or absence of different components rendered an intervention more or less effective (20). In addition, no statistically significant results were found for an association among intervention modality, duration, and outcomes (21), and the quality of the evidence was rated low.

The limitations included lack of methodological rigor (i.e., lack of reported data, small sample size, etc.), substantial heterogeneity, lack of clear description of included intervention components, lack of understanding of which intervention modality is most effective in decreasing caregiver psychological distress, and lack of consensus regarding most relevant outcomes and optimal intervention duration (20, 21).

The literature for both broad intervention categories highlights similar types of limitations. Both categories call for a better quality of studies that cover areas such as understanding which intervention modality is most effective in decreasing psychological distress, better classifications of interventions modalities, whether group or individual format is most effective, which intervention components are needed to show effect, and whether there is an optimal duration time (8, 13–15, 17, 20, 21). The call for better classifications of intervention modalities and understanding of intervention components that are needed to show effect is concerned with the theoretical understanding of what is creating the stressors that informal caregivers are experiencing. For example, The Pearlin Stress Process Model assumes a multidimensional approach of primary stress (e.g., care-receiver illness and symptoms of the illness) and secondary stress, (e.g., social, occupational, financial) (5, 22). It becomes crucial to understand what kind of outcome (e.g., depression, stress, anxiety, mastery, satisfaction with life, etc.) one is aiming to target when developing an intervention or investigating the effectiveness of an existing intervention.

In this systematic review and meta-analysis, we have attempted to address some of the abovementioned limitations. Our first aim was therefore to understand the effect of informal caregiver interventions, regardless of care-receiver mental illness or intervention modality (e.g., psychoeducational, multicomponent, technology, psycho-social etc.), on informal caregivers' symptoms of psychological distress compared to a waitlist, treatment as usual or active control group? Our second aim was to address some of the gaps in the literature by conducting several subgroup meta-analyses. We aimed to (1) investigate which intervention modality showed the most effect on decreasing caregiver psychological distress, (2) investigate which type of delivery format (group or individual) showed the most effect, (3) investigate which intervention components such as structure (manualized) and duration (≥8 weeks) and interventions including non-manualized interventions and ≤8 weeks showed the most effect, and (4) investigate the effect of informal caregiver interventions when grouped into two main categories (a) dementia/Alzheimer's disease and (b) severe mental disorders.

## Method

### Eligibility criteria

In conducting the systematic review and meta-analysis, we followed the PRISMA 2020 checklist (23). We included any randomized controlled trial (RCT) that included any intervention for adult informal caregivers of people with a mental disorder included in the *Diagnostic and Statistical Manual of Mental Disorders* [Fifth Edition] [*DSM-5*] (24) that was in real-time and taught by a mental health professional. The reason for only including RCTs in this review is that RCTs are considered the gold standard when it comes to assessing effect (16). We defined an informal caregiver as someone who is 18 years or older, a spouse, child, partner, parent, and/or other members of the family of a person with a mental illness. RCTs were included if they consisted of an outcome measure on psychological distress, depression, anxiety, stress, satisfaction with life, or emotion regulation. RCTs were included if the control group was either waitlist, treatment as usual, or active control. Next, RCTs were included if they were published in a peer-reviewed journal article in English. We excluded RCTs if the intervention was given in dyads (caregiver + care receiver), limited to the provision of respite care, and the patient sample included a mix of both physical and psychological illnesses. Finally, RCTs were excluded if they were unpublished, not peer reviewed, or study protocols.

### Identification of studies

We performed a search using the PubMed, PsycINFO, and Scopus databases for RCTs published from the first available year to June 2019, which reported on interventions for informal caregivers of people with a mental illness. The search terms used were: caregiver^*^ OR “carer” OR “informal caregiver^*^”) AND (interventions^*^ OR therapy OR “psycho-education” OR psychosocial^*^ OR “skills training” OR multicomponent OR group OR internet-based OR “stress reduction” OR “mindfulness training” OR “cognitive behavioral therapy” OR “CBT” OR “acceptance and commitment therapy” OR “ACT” OR “dialectical behavioral therapy” OR “DBT” OR “compassion training” OR psychological OR “support groups” OR “psychotherapy” OR “One-day” OR “face-to-face”) AND (“mental illness” OR “mental disorders” OR “schizophrenia” OR “anxiety disorders” OR “bipolar disorders” OR “post-traumatic stress disorders” OR “PTSD” OR depression^*^ OR “personality disorders” OR “mood disorders” OR “eating disorders” OR “obsessive compulsive disorders” OR “OCD” OR “attention deficit hyperactivity disorders” OR “ADHD” OR “autism spectrum disorders” OR Alzheimer's^*^ OR dementia^*^ OR “substance abuse disorders”) AND (randomis^*^ OR randomiz^*^ OR “controlled trial”) NOT (“literature review” OR “interview” OR “qualitative study” OR “meta-analysis” OR “systematic review” OR book^*^). The references of the selected studies were checked for additional eligible ones. The following criteria were applied for the selection.

### Outcome and data collection

The primary outcome was psychological distress, which was measured using a multitude of self-report questionnaires. We chose the outcome that the study author had chosen as the primary outcome. If a primary outcome did not measure psychological distress but a secondary outcome did, we included this one instead. If multiple primary or secondary outcomes had been used, we chose the first one that the study author had written in the measurement section. If a study had used two different instruments to measure an outcome (e.g., a self-report questionnaire and an interview instrument), we used the self-report questionnaire. If the study author had not reported which measure was primary and/or secondary, we chose the first one written in the measurement section.

### Quality assessment of the included studies

We used the Cochrane Risk of Bias (RoB) tool to assess the risk of bias in each study included. Two of the authors (NHH and KJ) completed the risk of bias assessment independently. Any issues that arose were resolved by discussion. Each of the following domains was rated high, low, or unclear risk of bias. The domains that were rated included (1) random sequence generation, (2) allocation concealment, (3) blinding of participants and personnel, (4) blinding of outcome assessment, (5) incomplete outcome data, (6) selective reporting, and (7) other sources of bias (refer to **Table 3** for risk of bias assessment). We also included the Jadad scale score, which rated the quality of each RCT on five domains: (1) Was the study described as random? (2) Was the randomization scheme described and appropriate? (3) Was the study described as double blind? (4) Was the method of double blinding appropriate? and (5) Was there a description of dropouts and withdrawals? The scores ranged from 0 to 5, and RCTs with a score between 0 and 2 were rated as low-quality, and RCTs with a score between 3 and 5 were rated as high-quality (see **Table 4**).

### Data extraction

Searches of the literature were conducted using the search strategy in the above-specified databases. Titles and/or abstracts were placed in a database to eliminate duplicates. Titles and/or abstracts that were not relevant were discarded, and the remaining titles and/or abstracts were carefully reviewed to determine their relevance based on the inclusion and exclusion criteria. Reference lists were also searched to determine if there were additional studies that had been overlooked. Once the titles and/or abstracts had been sorted, the full text of the studies was retrieved and assessed by two members of the review team (NHH and LB). When a study presented insufficient data, the authors attempted to contact the study authors; some answered right away, others did not respond to our request, and others had retired or passed away. The two authors reviewed the relevant articles independently, and disagreements were discussed until a consensus was reached.

The following data were collected: intervention modality, mental illness, number of participants allocated, treatment duration, outcome measured, baseline, post and any follow-up mean value, standard deviations, outcome analyzed, and information for the assessment of the risk of bias. Data regarding the adverse effects of the interventions were not collected. Any missing data were requested from the study authors.

### Analysis strategy

We extracted means and standard deviations from the included studies at baseline and the last follow-up measure in each study. We estimated within-group changes with standard error (SE) using STATA version 16. Some of the RCTs used self-report measures where an increase in the measurement indicated an effect (e.g., quality of life). When this was the case, we reverse scored so that in all the RCTs a reduction was in favor of intervention. We exported the data as well as participant totals (N) included in each group for each RCT into Review Manager 5.3 (RevMan 5.3) and estimated an overall standardized mean difference (SMD) with 95% CI including all the studies, and some subgroup analyses. We performed a random effects analysis as there was high heterogeneity among the studies (69). We used the statistics *I*^2^ to assess for inconsistency. *I*^2^ presents the percentage of the variability in effect estimates due to heterogeneity of intervention effects suggesting that the variation in effect estimates is beyond chance (70). To investigate if high heterogeneity was associated with the quality of included RCTs, we conducted a sensitivity analysis and excluded RCTs of poor quality (e.g., unclear risk of bias on either sequence generation, allocation concealment, incomplete outcome data, or selective outcome reporting).

## Results

### Study selection

**Figure 1:**
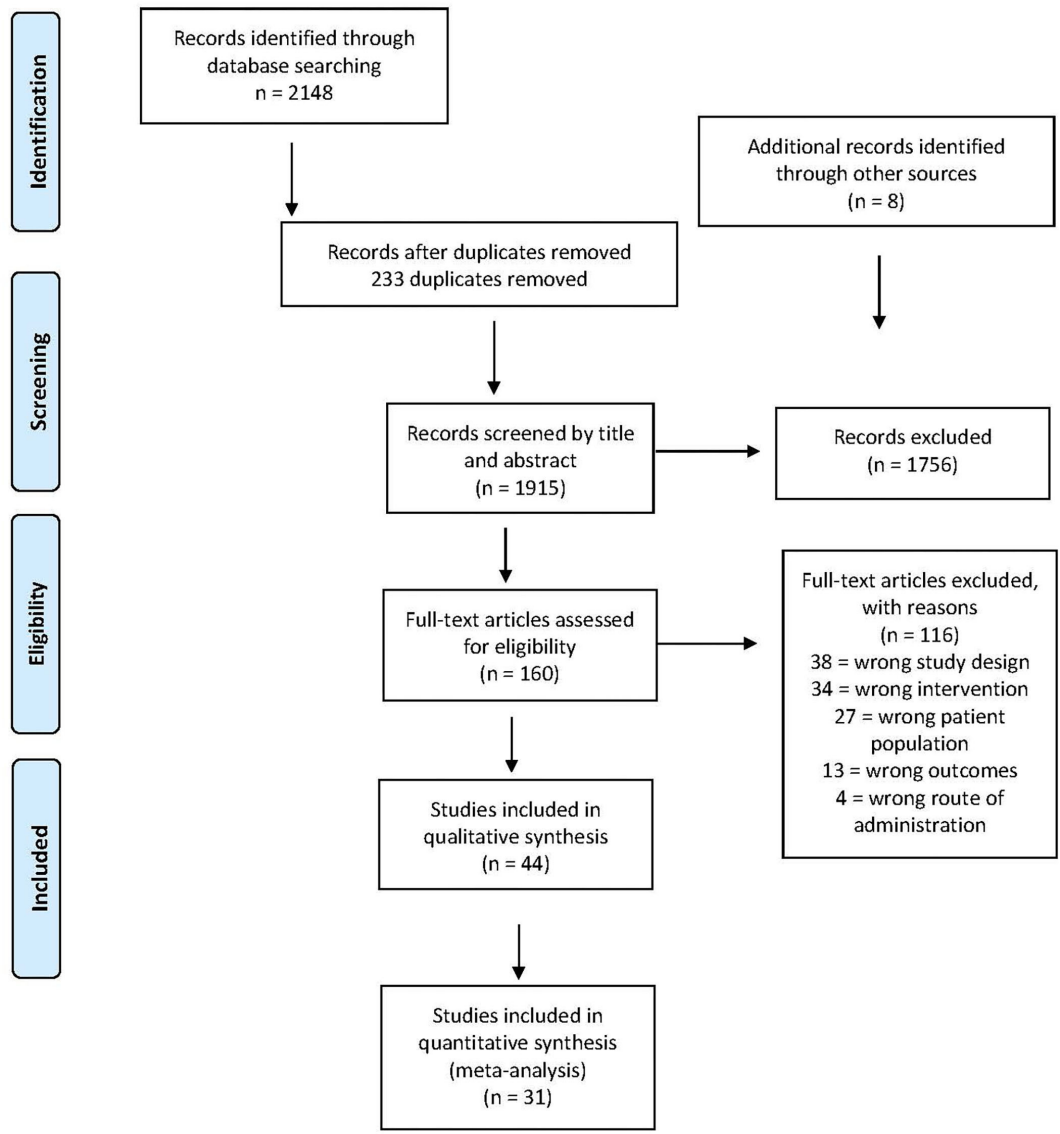
Prisma flow diagram.

The search yielded a total of 2,148 studies (Figure 1). There were 233 duplicates removed, leaving a total of 1,915 studies for title and abstract screening. We also went through the reference pages of the articles to make sure we had not left out any articles and found another eight articles, which we included. A total of 1,755 articles were excluded, and 160 full-text articles were assessed for eligibility. A total of 116 were excluded for the following reasons: not meeting the inclusion criteria for study design (*n* = 38), intervention (*n* = 34), patient population (*n* = 27), outcome (*n* = 13), and route of administration (*n* = 4) (Figure 1). In total, 44 studies were considered relevant and included in this review (Table 1). All 44 articles were assessed for quantitative data to be used in the meta-analysis. Of these, 13 studies could not be used because of insufficient data, leaving 31 studies to be included in the meta-analysis.

**Table 1:** Overview of all included RCTs.

**Reference**	**Study Population**	**Intervention**	**Control group**	**Measures**	**Follow-up**	**Reported conclusion**
Aakhus et al. (25)	Informal caregivers of psycho-geriatric in-patients	5 h non-manualized group psychoeducation *N* = 16	Waitlist *N* = 14	GHQ30, IES, GDS-30	End of intervention and 3 month follow-up	No group x time significant effect on self-reported psychological health
Akkerman and Ostwald (26)	Informal caregivers of people with Alzheimer's disease	9 week manualized group cognitive behavioral therapy *N* = 18	Waitlist *N* = 17	HAMA, BAI	End of intervention and 6 week follow-up	Group x time significant effect on self-reported anxiety
Ata and Dogan (27)	Informal caregivers of people with schizophrenia	7 week manualized individual cognitive behavioral stress management *N* = 28	Waitlist *N* = 33	ZCBS, COPE, GHQ-28, SIS	End of intervention	Group x time significant effect on self-reported burden
Barnes and Markham (28)	Informal caregivers of people with dementia	3 sessions manualized individual talking sense intervention *N* = 28	1 h session *N* = 27	HADS, ACQOL	3 month follow-up	No group x time significant effect on self-reported psychological health
Brown et al. (30)	Informal caregivers of people with Alzheimer's disease	8 week manualized mindfulness-based group intervention *N =* 23	8 week manualized social support group *N =* 15	PSS, POMS, SF-36, ZBI	End of intervention and 3 month follow-up	Group x time significant effect on self-reported stress at end of intervention
Berwig et al. (29)	Informal caregivers of people with Alzheimer's disease	6 month manualized individual multi-component intervention *N =* 47	Treatment as usual *N =* 45	ZBI, SF-12, RMBPC-24	End of intervention and 3 months follow-up	Group x time significant effect on self-reported burden
Cheng and Chan (31)	Informal caregivers of people with schizophrenia	10 week manualized psychoeducation group intervention *N =* 32	Treatment as usual *N =* 32	FBIS	End of intervention	Group x time significant effect on self-reported burden
Chou et al. (32)	Informal caregivers of people with schizophrenia	8 week manualized support group intervention *N =* 35	Waitlist *N =* 35	CBI, BDI	End of intervention and 1 month follow-up	Group x time significant effect on self-reported burden
Czaja et al. (33)	Informal caregivers of people with Dementia	6 month manualized individual multi-component intervention *N =* 38	(1) Received information *N =* 36 (2) Attention control intervention (Received same amount of contact) *N =* 36	RMBPC, CESD	End of intervention	Group x time significant effect on self-reported subjective burden
Danucalov et al. (34)	Informal caregivers of people with Alzheimer's disease	8 week manualized group multicomponent intervention *N =* 25	Waitlist *N =* 21	WHOQOL-BREF, MAAS, SCS	End of intervention	Group x time significant effect on self-reported quality of life
Davis et al. (35)	Informal caregivers of people with Dementia	3 month manualized technology-based intervention *N =* 24	Treatment as usual *N =* 22	CES-D, ZBI, SF-36	End of intervention	No group x time significant effect on self-reported depression
de Mamani and Suro (36)	Informal caregivers of people with schizophrenia	15 week manualized individual psychoeducation intervention *N =* 64	3 sessions of psychoeducational intervention *N =* 49	BAS	End of intervention	Group x time significant effect on self-reported subjective burden
de Souza et al. (37)	Informal caregivers of people with bipolar disorder	6 week non-manualized individual psychoeducational intervention *N =* 25	Treatment as usual *N =* 28	FBIS-BR, SF-36	End of intervention and 6 month follow-up	No group x time significant effect on self-reported subjective burden
Eisdorfer et al. (38)	Informal caregivers of people with Alzheimer's disease	(1) 12 month manualized individual psychosocial intervention *N =* 75 (2) 12 month manualized individual psychosocial intervention + technology support *N =* 73	12 month technology-based support intervention *N =* 73	CES-D, RMBPC	6 months follow-up	Group x time significant effect on self-reported depression in the multicomponent intervention (2)
Fung and Chien (39)	Informal caregivers of people with dementia	12 week manualized support group intervention *N =* 26	Treatment as usual *N =* 26	NPI, WHOQOL-BREF	End of intervention	Group x time significant effect on self-reported psychological health
Gallagher-Thompson et al. (40)	Informal caregivers of people with Dementia	4 month manualized individual psychoeducation intervention *N =* 23	4 month manualized individual technology-based intervention *N =* 22	CES-D-20, PSS, RMBPC-CB, SES	End of intervention	Group x time significant effect on self-reported depression
Garand et al. (41)	Informal caregivers of people with mild cognitive impairment and dementia	18 week manualized individual psychosocial intervention *N =* 36	18 week manualized individual nutritional training *N =* 37	CES-D, STAI	End of intervention, 1, 2, 6, and 12 month follow-up.	Group x time significant effect on self-reported depression and anxiety
Gendron et al. (42)	Informal caregivers of people with dementia	8 week manualized group cognitive behavioral therapy *N =* 18	8 week manualized information support group *N =* 17	Hopkins Symptom Checklist, Scale, BIS	End of intervention 3 and 6 month follow-up	No group x time significant effect on self-reported psychological health
Gerkensmeyer et al. (43)	Informal caregivers of children with mental health problems	8 week manualized individual technology-based intervention *N =* 30	Waitlist *N =* 31	BDI-II, Parent Experience Scale	End of intervention 3 and 6 month follow-up	No group x time significant effect on self-reported depression
Gonyea et al. (44)	Informal caregivers of people with Alzheimer's disease	5 week manualized group cognitive behavioral therapy + telephone coaching *N =* 33	5 week manualized group psycho-education + telephone coaching *N =* 34	NPI-D, CES-D, STAI-S	End of intervention 3 month follow-up	Group x time significant effect on self-reported psychological health.
Gonzalez et al. (45)	Informal caregivers of people with Alzheimer's disease	6 week non-manualized group multicomponent intervention *N =* 50	Treatment as usual *N =* 52	STAI, CES-D	End of intervention 3 month follow-up	No group x time significant effect on self-reported on self-reported anxiety
Gossink et al. (46)	Informal caregivers of people with dementia	6 month, manualized support group intervention *N =* 15	Treatment as usual *N =* 15	ZBI, PSS, CES-D	End of intervention	No group x time significant effect on self-reported subjective burden
Grover et al. (47)	Caregivers of people with anorexia nervosa	8 week manualized individual technology-based intervention *N =* 33	Treatment as usual *N =* 30	HADS, ECI	End of intervention 4 and 6 month follow-up	Group x time significant effect on self-reported depression
Caqueo-Urízar and Caqueo-Urízar (48)	Informal caregivers of people with schizophrenia	5 month manualized group psychosocial intervention *N =* 22	Treatment as usual *N =* 34	ZCBS	End of intervention	Group x time significant effect on self-reported subjective burden
Hubbard et al. (49)	Informal caregivers of people with bipolar disorder	2-session non-manualized group psychoeducation intervention *N =* 18	Waitlist *N =* 14	DASS, BAS	End of intervention 1 month follow-up	No group x time significant effect on self-reported subjective burden
Ji et al. (50)	Informal caregivers of people with Autism Spectrum Disorder	8 week manualized group multicomponent intervention *N =* 22	Waitlist *N =* 20	SF-36, CBI	End of intervention	Group x time significant effect on self-reported psychological health
Khoshknab et al. (51)	Informal caregivers of people with Schizophrenia	4 week manualized group psychoeducation intervention *N =* 36	Treatment as usual *N =* 35	FBIS	End of intervention 1 month follow-up	Group x time significant effect on self-reported subjective burden
Koolaee and Etemadi (52)	Informal caregivers of people with Schizophrenia	(1) 12 week manualized group psychosocial intervention *N =* 21 (2) 12 week manualized group psycho-education intervention *N =* 21	Treatment as usual *N =* 20	FBIS	End of intervention 3 and 6 month follow-up	Group x time significant effect on self-reported subjective burden
Lavretsky et al. (53)	Informal caregivers of people with Dementia	8 week manualized group and individual multicomponent intervention *N =* 23	8 week relaxation training *N =* 16	SF 36, HRSD	End of intervention	Group x time significant effect on self-reported psychological health
Leach et al. (54)	Informal caregivers of people with dementia	24 week manualized individual multicomponent intervention *N =* 8	Waitlist *N =* 9	HRQoL, WebNeuro	End of intervention and 3 month follow-up	No group x time significant effect on self-reported quality of life
Livingston et al. (55)	Informal caregivers of people with dementia	8 week manualized individual psychoeducation intervention *N =* 152	Treatment as usual *N =* 77	HADS, ZBI, COPE	End of intervention 4 and 8 month follow-up	Group x time significant effect on self-reported psychological health at the end of treatment
McCallion et al. (56)	Informal caregivers of children with developmental disabilities and delays	6-session non-manualized support group intervention *N =* 49	Waitlist *N =* 46	CES-D	3 month post intervention	Group x time significant effect on self-reported depression.
Mittelman et al. (57)	Informal caregivers of people with Alzheimer's disease	6-session non-manualized individual and group multicomponent intervention *N =* 199	Treatment as usual *N =* 197	OARS	4, 8, 12, 18, 24, month follow-up	Group x time significant effect on self-reported mental health
Mohide et al. (58)	Informal caregivers of people with dementia	6 month non-manualized individual psychoeducation intervention *N =* 30	Treatment as usual *N =* 30	CES-D, STAI, CQLI	End of intervention	No group x time significant effect on self-reported depression
Neece (59)	Informal caregivers of children with developmental delays	7 week manualized group mindfulness intervention	Waitlist *N =* 21	CES-D, SWLS, MASS, SCS	End of intervention	Group x time significant effect on self-reported depression
Oken et al. (60)	Informal caregivers of people with Dementia	(1) 7 week manualized group mindfulness intervention *N =* 10 (2) 7 weeks manualized group psychoeducation intervention *N =* 11	Respite *N =* 10	RMBPC, PSS, CES-D, MASS	End of intervention	Group x time significant effect on self-reported subjective burden in both interventions
Polo-Lopez et al. (61)	Informal caregivers of people with a mental disorder	10 week manualized individual cognitive behavioral therapy *N =* 29	Waitlist *N =* 20	SCL-90-R, STAI, BDI-II, SCQ	End of intervention and 6 month follow-up	Group x time significant effect on self-reported psychological health
Reinares et al. (62)	Informal caregivers of people with bipolar disorder	12 week manualized group psycho-education intervention *N =* 30	Waitlist *N =* 15	SBAS	End of intervention	Group x time significant effect on self-reported subjective burden
Stolley et al. (63)	Informal caregivers of people with Alzheimer's disease	4 h non-manualized individual psycho-education intervention *N =* 133	4 h, non-manualized individual information intervention *N =* 108	ZMBPC	3, 6, and 12 month follow-up	Group x time significant effect on self-reported subjective burden
Szmukler et al. (64)	Informal caregivers of people with Psychosis	9 months non-manualized individual therapy *N =* 26	Treatment as usual *N =* 23	CISR, ECI, COPI	End of intervention and 6 month follow-up	No group x time significant effect on self-reported psychological health
Szmukler et al. (65)	Informal caregivers of people with Schizophrenia	6 week manualized individual psychoeducation intervention *N =* 32	1 h information session *N =* 31	GHQ-28, ECI, Ways of Coping WOC	3 and 6 month follow-up	No group x time significant effect on self-reported psychological health
Tabeleão et al. (66)	Informal caregivers of people with mental disorders	6-session manualized individual psycho-education intervention *N =* 66	Treatment as usual *N =* 64	ZBI, SRQ-20 BHS	End of intervention	Group x time significant effect on self-reported subjective burden
Tonge et al. (67)	Informal caregivers of children with Autism	20 week manualized group psychoeducation + behavior management intervention *N =* 35	20 week manualized group psychoeducation + counseling intervention *N =* 35	GHQ-28. Parenting Stress Thermometer	End of intervention and 6 month follow-up	Group x time significant effect on self-reported psychological health
Whitebird et al. (68)	Informal caregivers of people with dementia	8 week manualized mindfulness group intervention *N =* 38	8 week manualized group psychoeducation + social support intervention *N =* 40	PSS, CES-D, STAI, SF-12	End of intervention and 6 month follow-up	Group x time significant effect on self-reported stress at the end of intervention

### Overview of the findings of the systematic review

An overview of all the included RCTs is presented in Table 1. The intervention group significantly improved mental health compared to a waitlist, treatment as usual, or active control group on at least one self-reported outcome in thirty-one of the forty-four included studies. An effect is seen in psychoeducation, psychosocial, multicomponent, cognitive behavioral therapy, and mindfulness-based and support group interventions. The no-effect studies are characterized by short duration, having a long-term follow-up period, or being a technology-based intervention. Furthermore, twenty-three studies investigated the effectiveness of informal caregiver interventions in people with dementia or Alzheimer's disease, and psychological distress was decreased in 16 RCTs. Four out of five individual psychoeducational interventions decreased psychological distress. All three mindfulness-based interventions decreased psychological distress. Five out of seven multicomponent interventions decreased psychological distress. Four out of six psychosocial or support group interventions decreased psychological distress, whereas neither individual therapy nor technology-based intervention decreased psychological distress. Moreover, 21 RCTs were included in the category of interventions for informal caregivers of people with severe mental illness, and 15 decreased psychological distress. Six out of ten psychoeducational interventions decreased psychological distress. All five psychosocial or support group interventions decreased psychological distress. One mindfulness intervention and one multicomponent intervention decreased psychological distress, whereas one out of two individual therapy interventions and one technology-based intervention decreased psychological distress.

Regarding the specific outcome measures of psychological distress included in the RCTs, Table 2 reflects the mean difference in change from baseline in all the specific outcome measures of psychological distress used in the meta-analysis. Some of the results in Table 2 show a non-significant effect on the outcome measure, while the study author has reported a significant effect on the outcome measure. This discrepancy between the two results is most likely due to the different statistical models used. We report here both the results found in the meta-analysis and the results reported by the study author. Therefore, two RCTs showed effect on depression (40, 56), two RCTs showed effect on anxiety (26, 55), three RCTs showed effect on stress (30, 60, 68), four RCTs showed effect on the quality of life (34, 50, 53, 67), seven RCTs showed effect on subjective burden (27, 29, 48, 51, 52, 62, 66), and three RCTs showed effect on psychological distress (39, 44, 61).

**Table 2:** Mean difference in change from baseline in the specific outcomes of psychological distress included in the meta-analysis.

**Outcome**	**RCT**	**Intervention**	**Control group**	**Mean difference in change from baseline with 95% CI**
Depression	Davis et al. (35)	Technology	Treatment as usual	CES-D 0.29 (-8.76, 9.34)
	Gallagher-Thompsen et al. (40) ^*^	Psychoeducation	Technology	CES-D -5.40 (-13.95, 3.15)
	Hubbard et al. (49)	Psychoeducation	Waitlist	DASS 1.39 (-1.05, 3.83)
	McCallion et al. (56)^*^	Support Group	Waitlist	CES-D -4.80 (-10.61, 1.01)
	Mohide et al. (58)	Psychoeducation	Treatment as usual	CES-D -0.18 (95% CI−0.78 to 0.43)
Anxiety	Akkerman and Ostwald (26)	Cognitive Behavioral Therapy	Waitlist	BAI -10.49 (-17.79,−3.19)
	Gonzales et al. (45)	Multicomponent	Treatment as usual	STAI 2.36 (-4.32, 9.04)
	Livingston et al. (55)^*^	Psychoeducation	Treatment as usual	HADS -0.69 (-3.59, 2.21)
Stress	Brown et al. (30) ^*^	Mindfulness	Social Support	PSS 0.05 (-0.82, 0.92)
	Whitebird et al. (68)^*^	Mindfulness	Psychoeducation + Social Support	PSS -2.70 (-6.65, 1.25)
	Oken et al. (60)^*^	Mindfulness	1.Psychoeducation 2.Respite	RMBPC 4.20 (-9.52, 17.92)
Subjective burden	Ata and Dogan (27)	Cognitive behavioral stress management	Waitlist	ZCBS -15.16 (-25.45,−4.87)
	Berwig et al. (29)	Multicomponent	Treatment as usual	ZBI -5.43 (-9.78,−1.08)
	de Souza et al. (37)	Psychoeducation	Treatment as usual	FBIS 1.10 (-8.65, 10.85)
	Gossink et al. (46)	Support Group	Treatment as usual	ZBI -1.40 (-9.61, 6.81)
	Gutiérrez-Maldonado and Caqueo-Urízar (48)	Psychosocial	Treatment as usual	ZCBS -32.19 (-43.66,−20.72)
	Khoshknab et al. (51)	Psychoeducation	Treatment as usual	FBIS -16.13 (-18.01,−14.25)
	Koolae et al. (52)	Psychosocial	Treatment as usual	FBIS -17.52 (-26.37,−8.67)
	Reinares et al. (62)^*^	Psychoeducation	Waitlist	SBAS -0.17 (-0.39, 0.05)
	Tabeleáo et al. (66)	Psychoeducation	Treatment as usual	ZBI -2.90 (-9.97, 4.17)
Quality of life	Aakhus et al. (25)	Psychoeducation	Waitlist	GHQ 6.95 (0.03, 13.87)
	Danucalov et al. (34)	Multicomponent	Waitlist	WHOQOL- BREF -3.00 (-5.35,−0.65)
	Ji et al. (50)^*^	Multicomponent	Waitlist	SF-36 -3.01 (-13.32, 7.30)
	Lavretsky et al. (53)	Multicomponent	Relaxation	SF-36 -2.10 (-4.77, 0.57)
	Leach et al. (54)	Multicomponent	Waitlist	AQoL-8D 0.03 (-0.10, 0.16)
	Szmukler et al. (65)	Psychoeducation	Information	GHQ-28 4.90 (-4.03, 13.83)
	Tonge et al. (67)^*^	Psychoeducation + behavior management	Psychoeducation + counseling	GHQ-28 -6.12 (-13.14, 0.90)
Psychological distress	Fung and Chien (39)^*^	Support Group	Treatment as usual	NPI -5.02 (-13.90, 3.86)
	Gendron et al. (42)	Cognitive behavioral Therapy	Information Support	HSCL -0.40 (-5.94, 5.14)
	Gonyea et al. (44)^*^	Cognitive behavioral Therapy	Psychoeducation	NPI, -0.60 (-4.91, 3.71)
	Polo-Lopez et al. (61)^*^	Cognitive behavioral Therapy	Waitlist	SCL-90, -1.26 (-10.67, 8.15)
	Szmukler et al. (64)	Individual therapy	Treatment as usual	CISR -2.80 (-10.14, 4.54)

* Those RCTs where the study author reported significant effect of the outcome measure. BAI, Becks Anxiety Inventory (71); STAI, State Trait Anxiety Inventory (72); DASS, Depression Anxiety Stress Scale (73); RMBPC, The Revised Memory and Behavior Problem Checklist (74); BAS, The Modified Burden Assessment Scale (75); WHOQOL-BREF, World health Organization Quality of Life Questionnaire (76); ZCBS, Zarit Caregiver Burden Scale (77); MBPC., Zarit Memory and Behavior Problems Checklist (78); CISR, Clinical Interview Schedule Revised (79); HSCL, Hopkins Symptom Checklist (80); SCL-90 Symptom Check List-90 (80); AQoL-8D- Assessment of Quality of Life 8-Dimension (81).

Lastly, according to Cochrane's Risk of Bias, all 44 studies included were of low quality (Table 3). A total of 28 RCTs had low and 16 had unclear risk of bias on sequence generation. A total of 32 had low and twelve had unclear risk of bias on allocation concealment. A total of 37 had a high risk of bias, 4 unclear, and 3 low on blinding of participants and personnel. A total of 19 RCTs were unclear, 18 low, and 7 high on blinding of outcome assessors. A total of 39 had low, four had unclear, and 1 had a high risk of bias on incomplete data. A total of 22 had low and 22 had unclear risk of bias on selective outcome reporting. According to the Jadad quality score, 21 of the 44 studies were of good quality (Table 4).

**Table 3:** Cochranes Risk of Bias.

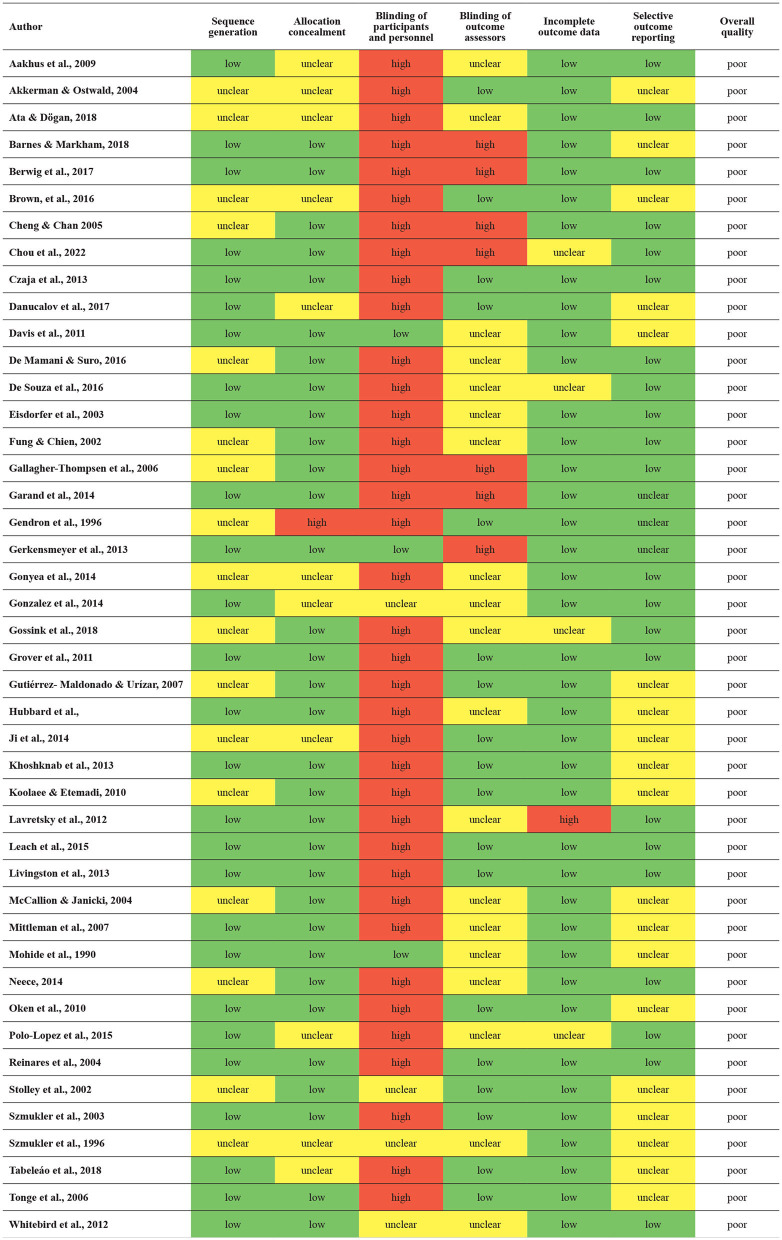

Green indicates low risk of bias, yellow indicates unclear risk of bias, and red indicates high risk of bias.

**Table 4:** Jadad quality score.

**Reference**	**Was the study described as random**	**Was the randomization scheme described and appropriate?**	**Was the study described as double-blind?**	**Was the method of double blinding appropriate?**	**Was there a description of dropouts and withdrawals?**	**Total Jadad score**
Aakhus et al. (25)	1	1	0	0	1	3
Akkerman and Ostwald (26)	1	0	0	0	1	2
Ata and Dogan (27)	1	0	0	0	1	2
Barnes and Markham (28)	1	1	0	0	1	3
Berwig et al. (29)	1	1	0	0	1	3
Brown et al. (30)	1	0	0	0	1	2
Cheng and Chan (31)	1	0	0	0	1	2
Chou et al. (32)	1	1	0	0	0	2
Czaja et al. (33)	1	1	0	0	1	3
Danucalov et al. (34)	1	1	0	0	1	3
Davis et al. (35)	1	1	0	0	1	3
de Mamani and Suro (36)	1	0	0	0	1	2
de Souza et al. (37)	1	1	0	0	0	2
Eisdorfer et al. (38)	1	1	0	0	1	3
Fung and Chien (39)	1	0	0	0	1	2
Gallagher-Thompsen et al. (40)	1	0	0	0	1	2
Garand et al. (41)	1	1	0	0	1	3
Gendron et al. (42)	1	0	0	0	1	2
Gerkensmeyer et al. (43)	1	1	0	0	1	3
Gonyea et al. (44)	1	0	0	0	1	2
Gonzalez et al. (45)	1	1	0	0	1	3
Gossink et al. (46)	1	0	0	0	0	1
Grover et al. (47)	1	1	0	0	1	3
Gutiérrez-Maldonado and Caqueo-Urízar (48)	1	0	0	0	1	2
Hubbard et al. (49)	1	1	0	0	1	3
Ji et al. (50)	1	0	0	0	1	2
Khoshknab et al. (51)	1	1	0	0	1	3
Koolaee and Etemadi (52)	1	0	0	0	1	2
Lavretsky et al. (53)	1	1	0	0	1	3
Leach et al. (54)	1	1	0	0	1	3
Livingston et al. (55)	1	1	0	0	1	3
McCallion et al. (56)	1	0	0	0	1	2
Mittleman et al. (57)	1	1	0	0	1	3
Mohide et al. (58)	1	1	0	0	1	3
Neece (59)	1	0	0	0	1	2
Oken et al. (60)	1	1	0	0	1	3
Polo-Lopez et al. (61)	1	1	0	0	0	2
Reinares et al. (62)	1	1	0	0	1	3
Stolley et al. (63)	1	0	0	0	1	2
Szmukler et al. (64)	1	1	0	0	1	3
Szmukler et al. (65)	1	0	0	0	1	2
Tabeleão et al. (66)	1	1	0	0	1	3
Tonge et al. (67)	1	1	0	0	1	3
Whitebird et al. (68)	1	1	0	0	1	3

### Overview of the results of the meta-analysis

**Figure 2:**
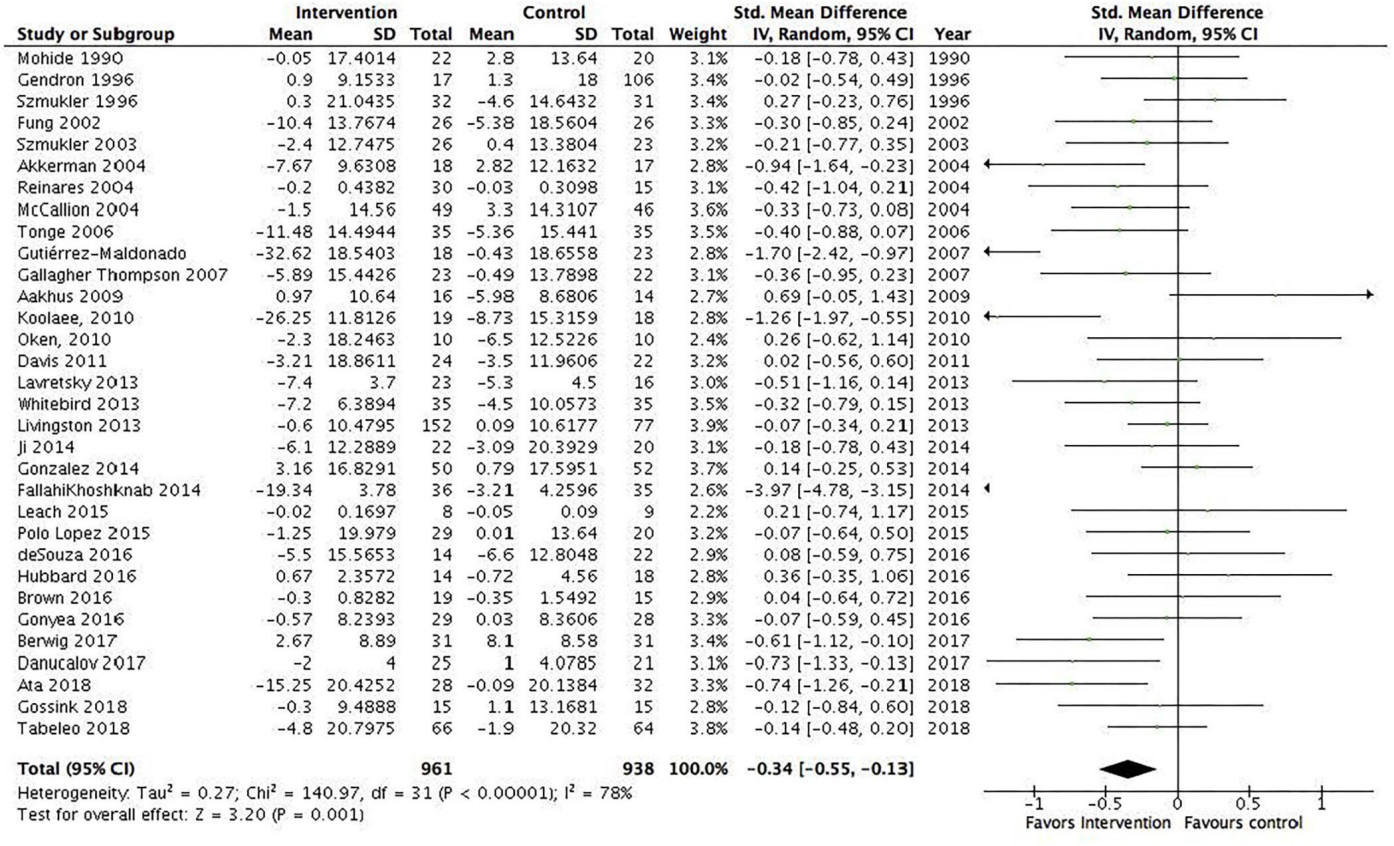
All informal caregiver interventions included in the meta-analysis. Mean within-group changes and standardized mean differences.

**Figure 3:**
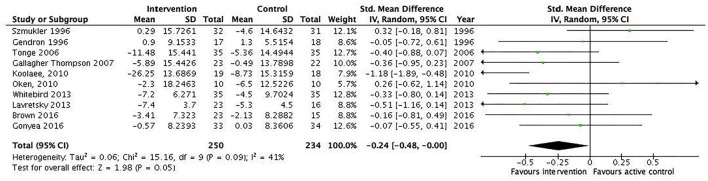
Subgroup analysis of all informal caregiver interventions that included an active control group. Mean within-group changes and standardized mean differences.

A total of 31 RCTs (*N* = 1,899) were included in the meta-analysis. We found a statistically significant pooled effect favoring the intervention over waitlist, treatment as usual, or active control: −0.32 (95% CI −0.53 to −0.11) (Figure 2). *I*^2^ was 78%, suggesting a substantial heterogeneity among the included RCTs. The sensitivity analysis with the exclusion of RCTs with poor quality resulted in an *I*^2^ of 89%, suggesting an even more substantial heterogeneity among the included RCTs (Supplementary Figure 1). Ten RCTs were included in the sensitivity analysis. Five RCTs showed no effect, and the majority of RCTs were characterized by short duration and being a technology-based, psychoeducational, multi-component, or transcendental meditation intervention. Furthermore, a total of 10 RCTs were included in the meta-analysis with an active control group (10 studies, *N* = 484). We found a statistically significant pooled effect estimate favoring the intervention compared to an active control group: −0.24 (95% CI: −0.48 to 0) (Figure 3). *I*^2^ was 41%, suggesting a moderate heterogeneity. In this analysis, three of the included RCTs compared mindfulness-based interventions (MBIs) with psychoeducation, psychoeducation + support, or social support, and the results showed that two of the MBIs were more effective in decreasing psychological distress than the active intervention and that one MBI was as effective as a psychoeducation + support intervention. Two RCTs compared a psychosocial intervention with a technology-based and a psychoeducational intervention and found an effect for the psychosocial intervention. Two RCTs found an effect for a psychoeducational intervention compared to a technology-based or information intervention. Two RCTs found an effect for a CBT intervention compared to either psychoeducation or information interventions, and one RCT found an effect for a multicomponent intervention compared to a relaxation intervention. The sensitivity analysis with the exclusion of RCTs with poor quality resulted in an *I*^2^ of 0%, suggesting no heterogeneity among the included RCTs (Supplementary Figure 1). The sensitivity analysis included two RCTs characterized by being 8 week manualized group mindfulness and yoga-and-compassion-based interventions. The moderate heterogeneity may have been due to the low quality of the included RCTs.

**Figure 4:**
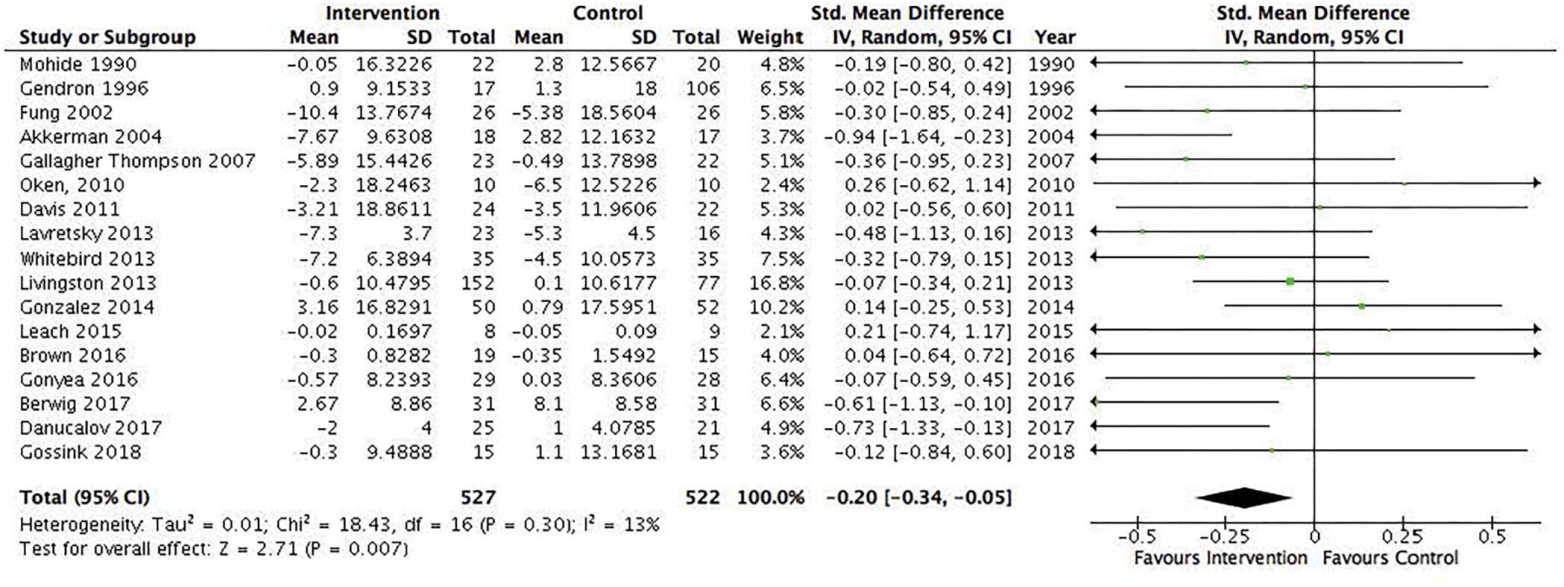
Subgroup analysis of interventions for informal caregivers of people with dementia/Alzheimer's disease, individual/group: All Studies. Mean within-group changes and standard.

**Figure 5:**
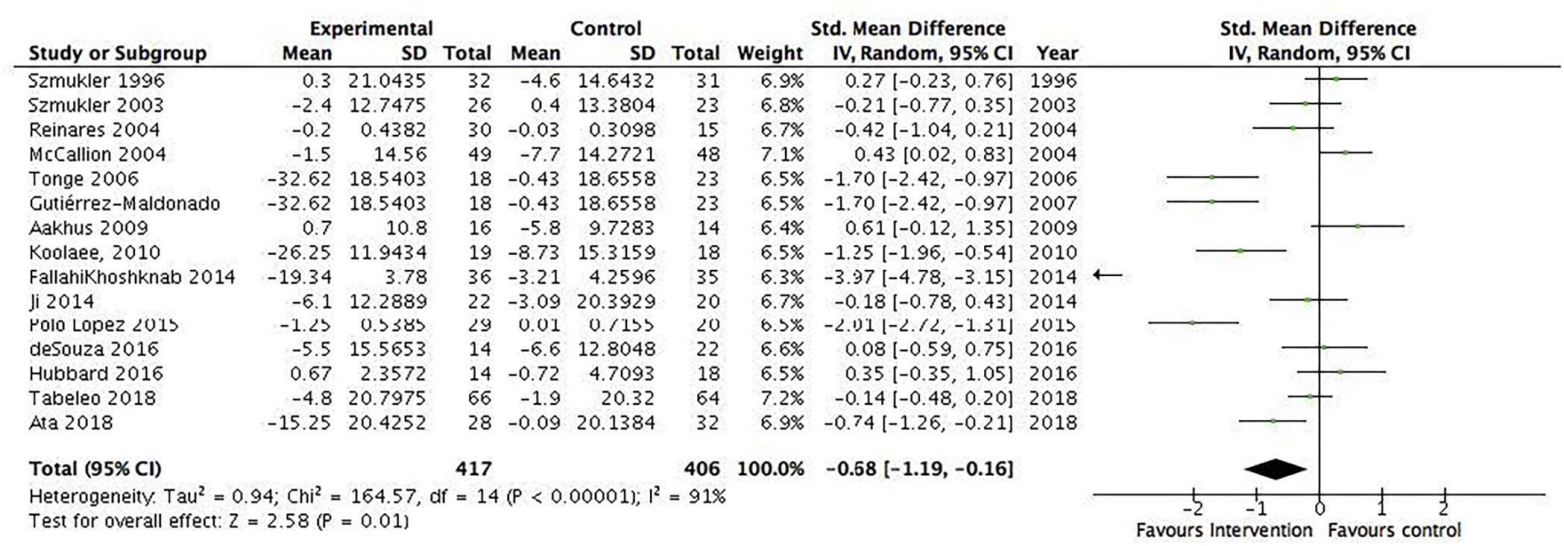
Subgroup analysis of interventions for informal caregivers of people with severe mental illness, group/individual: All studies. Mean within-group changes and standardized mean differences.

**Figure 6:**
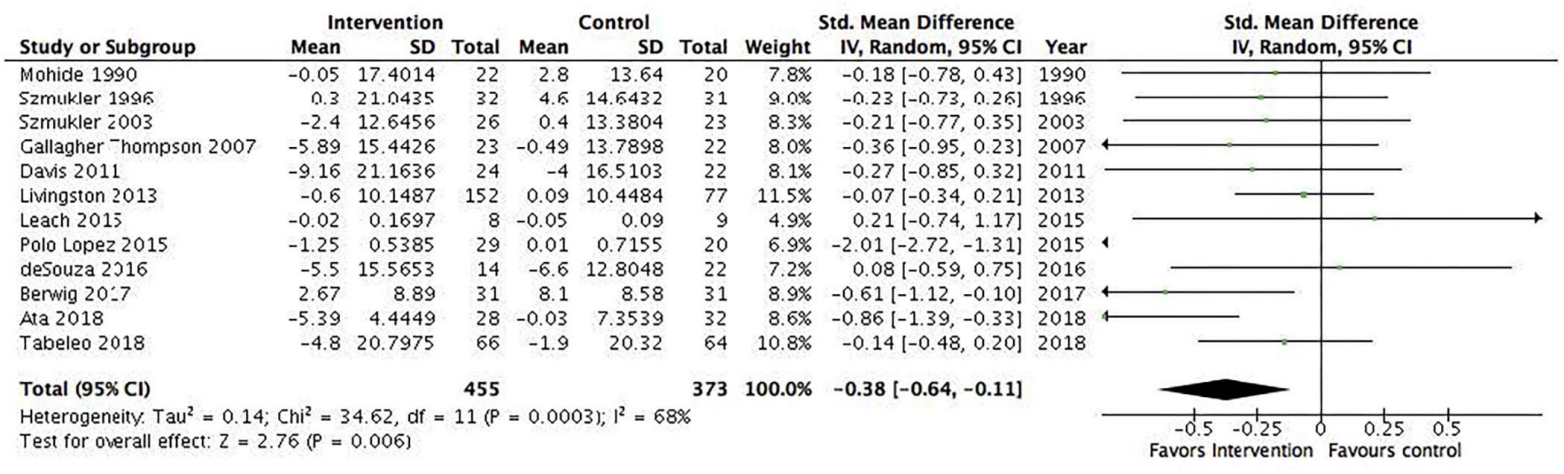
Subgroup analysis of interventions with an individual delivery format: All studies. Mean within-group changes and standardized mean differences.

**Figure 7:**
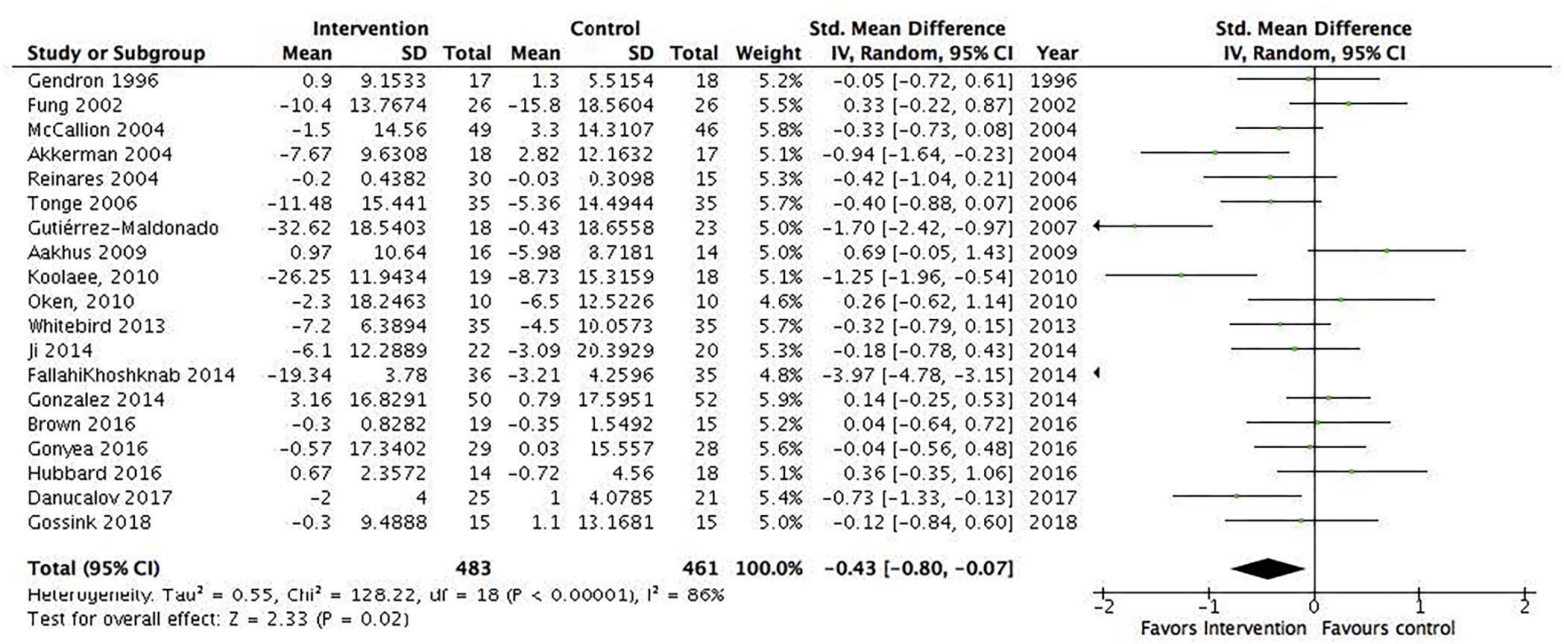
Subgroup analysis of interventions with a group delivery format: All studies. Mean within-group changes and standardized mean differences.

**Figure 8:**
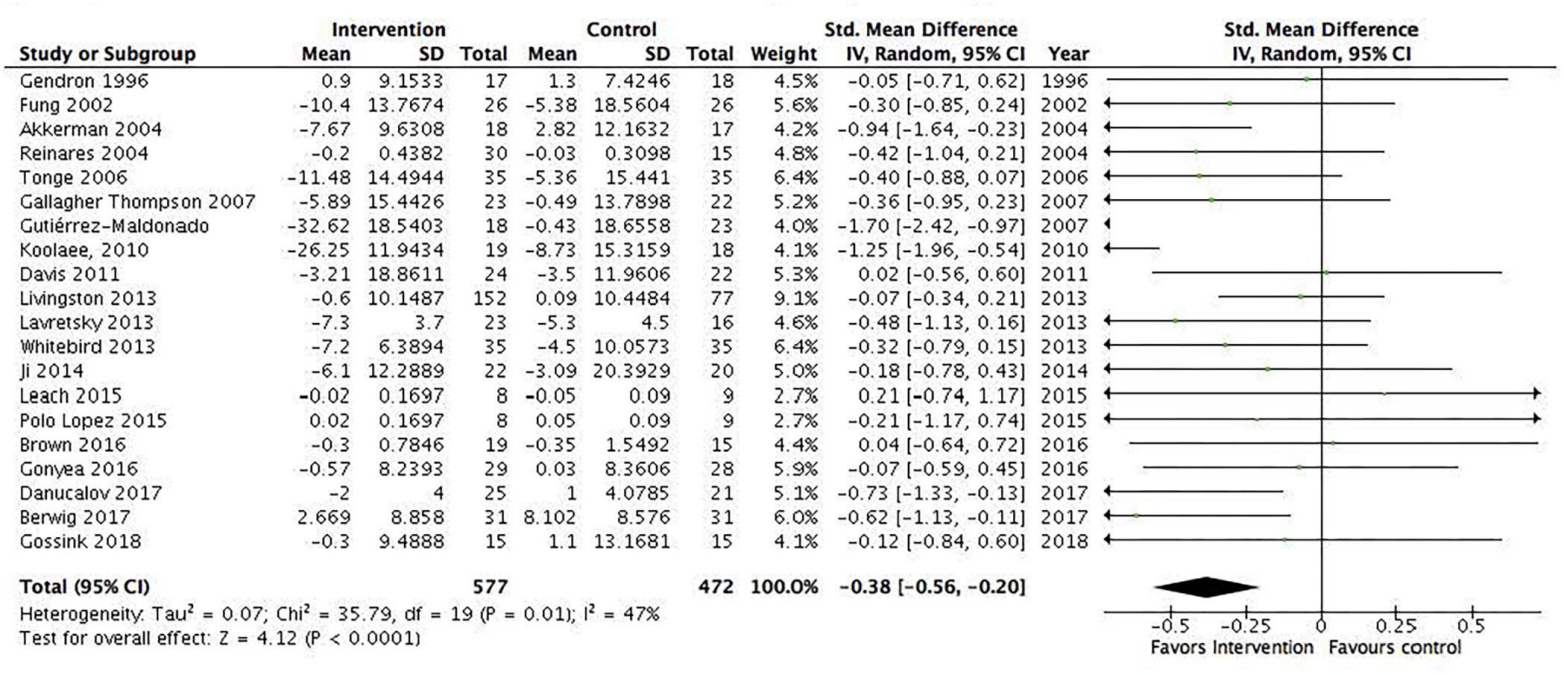
Subgroup analysis of interventions that were manualized and ≥8 weeks duration, group/individual: All studies. Mean within-group changes and standardized mean differences.

**Figure 9:**
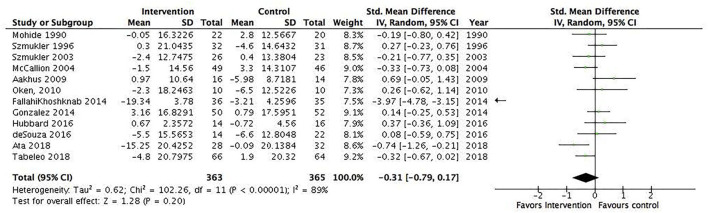
Subgroup analysis of interventions that were non-manualized interventions, ≤8 weeks of duration, individual/group delivery format: All studies. Mean within-group changes and standardized mean differences.

Several subgroup analyses were conducted. The first included interventions for informal caregivers of people with dementia/Alzheimer's disease (17 studies, *N* = 1,049) and showed a statistically significant pooled effect favoring interventions for informal caregivers of people with dementia/Alzheimer's disease, −0.2 (95% CI: −0.34 to −0.05) (Figure 4). The *I*^2^ was 13%, suggesting a low heterogeneity between the included RCTs. The second included all interventions for informal caregivers of people with a severe mental illness (15 studies, *N* = 823) and showed a statistically significant pooled effect favoring interventions for informal caregivers of people with a mental illness, −0.68 (95% CI: −1.19 to −0.16) (Figure 5). The I^2^ was 91%, suggesting a high heterogeneity among the included RCTs. The sensitivity analysis with the exclusion of RCTs with poor quality resulted in an *I*^2^ of 96%, suggesting an even more substantial heterogeneity among the included RCTs (Supplementary Figure 1). The third subgroup analysis included all interventions with an individual delivery format (12 studies, *N* = 828) and showed a statistically significant pooled effect favoring individual interventions, −0.38 (95% CI: −0.64 to −0.11) (Figure 6). The *I*^2^ was 68%, suggesting a high heterogeneity among the included RCTs. The sensitivity analysis with the exclusion of RCTs with poor quality resulted in an *I*^2^ of 4%, suggesting no heterogeneity among the included RCTs (Supplementary Figure 1). Five RCTs were included in the sensitivity analysis. One RCT carried the main effect and was characterized by having a duration of 10 sessions being an individual manualized multicomponent intervention, while one RCT was underpowered with small sample size and consisted of a transcendental meditation program. The substantial heterogeneity was most likely due to the quality of the included RCTs. The fourth subgroup analysis included all interventions with a group delivery format (19 studies, *N* = 932) and showed a statistically significant pooled effect favoring group interventions, −0.43 (95% CI: −0.8 to −0.07) (Figure 7). The *I*^2^ was 86%, suggesting there was substantial heterogeneity among the included RCTs. The sensitivity analysis with the exclusion of RCTs with poor quality resulted in an *I*^2^ of 95%, suggesting an even more substantial heterogeneity among the included RCTs (Supplementary Figure 1). The fifth subgroup analysis included all manualized interventions that were ≥8 weeks long and had either an individual or group delivery format (20 studies, *N* = 1,049). The results showed a statistically significant pooled effect favoring interventions ≥8 weeks long and manualized, −0.38 (95% CI: −0.56 to −0.2) (Figure 8). The *I*^2^ was 47%, suggesting a moderate heterogeneity among the included RCTs. The sensitivity analysis with the exclusion of RCTs with poor quality resulted in an *I*^2^ of 2%, suggesting a low heterogeneity among the included RCTs (Supplementary Figure 1). Seven RCTs were included in the sensitivity analysis. Of these, two carried the main effect and were characterized by a group CBT and an individualized multicomponent intervention. The moderate heterogeneity may be due to the quality of the included RCT. The sixth subgroup analysis included all non-manualized interventions that were <8 weeks long and had either an individual or a group delivery format (21 studies, *N* = 728). The results did not show a statistically significant pooled effect favoring interventions ≤8 weeks long and non-manualized, −0.31 (95% CI: −0.79 to 0.17) (Figure 9). The *I*^2^ was 89%, suggesting a substantial heterogeneity among the included RCTs. The sensitivity analysis with exclusion of RCTs with poor quality resulted in an *I*^2^ of 96%, suggesting an even more substantial heterogeneity among the included RCTs (Supplementary Figure 1).

## Discussion

The results of the systematic review showed that thirty-one of the forty-four RCTs showed an effect of the intervention decreasing informal caregiver psychological distress. This was true for both broad categories (dementia/Alzheimer's disease and severe mental illness) as well as for interventions delivered in individual and group formats. An effect was seen for psychoeducation, psychosocial, multicomponent, cognitive behavioral therapy, mindfulness-based, and support group interventions. While we did not evaluate the remaining thirteen RCTs quantitatively because of lack of effect, they appeared to be characterized by being of short duration, including long-term follow-up, and being technology-based interventions. All the RCTs were of poor quality according to Cochrane's R of B, but twenty-one of the RCTs had a good quality according to the Jadad quality score.

Thirty-one studies had sufficient data to conduct a meta-analysis including subgroup analysis (*N* = 1,899). Of the RCTs that were included in the meta-analysis and that showed an effect, two measured depression, two measured anxiety, three stress, four quality of life, seven subjective burden, and three measured psychological distress. The results of the meta-analysis showed a small statistically significant effect (−0.32) of informal caregiver interventions on psychological distress compared to waitlist, treatment as usual, or active control regardless of care-receiver mental illness or intervention modality. Moreover, the results suggest that many different intervention modalities show an effect on decreasing informal caregiver's psychological distress. However, most of the studies only measured psychological distress by the end of the intervention, and there was a high heterogeneity among the RCTs except for interventions for informal caregivers of people with dementia/Alzheimer's disease (*I*^2^ = 13%), manualized, at least 8 weeks duration, group/individual delivery format (*I*^2^ = 43%), and RCTs that compared interventions with an active control group (*I*^2^ = 41%).

The results of the two subgroup analyses, which included interventions for informal caregivers of people with dementia/Alzheimer's disease, showed a small effect (−0.18), while the interventions for informal caregivers of people with a severe mental illness showed a moderate effect v.68). The apparent difference in effect size between the two broad categories begs the question whether it makes sense to conduct this type of subgroup analysis. While the dementia subgroup consisted of almost the same number of studies (17) as the severe mental illness subgroup (15), the heterogeneity of the dementia group was much smaller than the heterogeneity of the severe mental illness group. The results indicate that the RCTs in the dementia subgroup are more similar than the RCTs included in the severe mental illness group, and that the results of the dementia subgroup analysis may be closer to reality than those of the severe mental illness group. We should therefore be careful when interpreting the results of the subgroup analysis. Results of the subgroup analysis investigating interventions with an individual delivery format compared to waitlist, treatment as usual or active control showed a small effect (−0.38), and so did group delivery format (−0.43). The results of the subgroup analysis that included interventions that were manualized, at least 8 weeks duration, and had either an individual or a group delivery format compared to waitlist, treatment as usual or active control showed a small effect (−0.38), while the results of the subgroup analysis that included RCTs with non-manualized interventions of less than 8 weeks duration compared to waitlist, treatment as usual or active control showed no effect. The sensitivity analysis of the meta-analysis including all the RCTs showed that when low quality studies were excluded the heterogeneity increased. The same happened for the subgroup analysis of group delivery format, severe mental illness and non-manualized, <8 weeks, and individual/group. This suggests that the substantial heterogeneity was due to different intervention modalities, durations, and structures being pooled together. The sensitivity analysis for the subgroups, individual delivery format, manualized, 8 weeks or more, group/individual, and active control showed that when low-quality RCTs were excluded the heterogeneity was decreased.

Taken together, while the meta-analysis showed a small effect on informal caregiver psychological distress regardless of intervention modality, delivery format, and care-receiver mental illness, the subgroup and sensitivity analyses suggest that only some interventions are effective. The effect seems to be carried by a few high-quality RCTs characterized by being manualized, duration, and with active participation (e.g., mindfulness-based and cognitive-behavioral-based interventions). Thus, the intervention components, duration, and structure (manual) are important to improve caregivers' mental health. It may be important to continue to investigate the optimal number of sessions, as the literature is both lacking and presenting conflicting evidence (18, 19). It may also be important to think about the duration of an intervention when decisions regarding the program theory and when developing an intervention or using an existing one are being made.

We overcame some limitations of previous reviews by only including RCTs, adding to the methodological rigor, and we replicated findings from 3 decades ago (8, 82), showing a small effect of informal caregiver interventions on psychological distress. Moreover, as a previous meta-analysis has suggested (13), we found that structured (manual) interventions with active participation and eight or more sessions (≥8 weeks) had the best effect. We were not able to investigate which intervention modality showed the most effect on decreasing caregiver psychological distress, as it was very difficult to classify the interventions as different components were mixed together within interventions. This problem has also been reported in other systematic reviews and meta-analyses (14, 20, 83). However, the results of the subgroup analysis including interventions compared with an active control showed a small effect (−0.24). When two interventions were directly compared, mindfulness-based interventions were superior to psychoeducation. Psychoeducation with active participation was superior to psychoeducation without active participation. Moreover, psychosocial and psychoeducational interventions were superior to technology-based interventions. CBT interventions were superior to both psychoeducational and informational interventions, and multicomponent interventions were superior to relaxation interventions.

Our findings in this systematic review and meta-analysis echo the general recommendations of previous systematic review and meta-analysis within the field. There is a great need to have clearly defined intervention categories (14) and to clearly specify the different intervention components included and what outcomes the different components aim to target. For instance, psychoeducational interventions are characterized by having a structured program toward providing information about the care-receivers' disease and how to respond effectively to the illness-related problems, with support being secondary to the educational component (8, 17). Supportive interventions are characterized by being either professionally or peer-led and unstructured while building rapport and creating a safe place for caregivers to share their concerns. Multicomponent interventions are characterized by inclusion of a combination of support, psychotherapy, and educational components (8), and psychosocial interventions have been defined as interventions that emphasize psychological or social factors (84). While the abovementioned categorizations appear to make sense, the literature is presenting a different picture.

The issue with these categories is that they do not consider the program theory underlying the development of the different types of interventions. Perhaps it would be better to classify interventions based on their program theory and the outcome they are developed to target. In addition, when authors do not explicitly present the different components included in the intervention, it becomes a subjective decision of the authors conducting systematic reviews and meta-analyses to decide in which category the different interventions should be placed (14, 21, 83). Moreover, there is little consideration for the mechanisms of the intervention creating the wanted effect. As an example, one systematic review attempted to explore the relationship between intervention content and outcome. The categorizations included (1) psychoeducation only, (2) psychoeducation plus mutual support, and (3) psychoeducation plus skills training. While the results suggested that there was a positive effect of interventions on at least one outcome, there was no evidence that the presence or absence of any of the key components made an intervention more or less effective (20). It would be helpful to conduct systematic reviews that include other types of research design (i.e., qualitative studies) where the main aim is to investigate mechanisms of change.

As has been reported elsewhere (20, 21, 82), most of the subgroup analyses showed a small effect, presence of substantial heterogeneity, small sample sizes, and many inconsistencies regarding outcome measures. In the PROSPERO protocol, we divided the outcome measures into primary (psychological distress) and secondary outcomes (anxiety, depression and perceived stress, subjective wellbeing, and emotion regulation). While carefully reading the included studies, it became clear that the outcome measures used to measure psychological distress were not similar. This finding is also consistent with previous research in this field (14, 82); therefore, we decided to broadly define psychological distress to include depression, subjective burden, anxiety, and perceived stress (82). It is possible that we would have seen different results had we conducted a subgroup analysis based on interventions that used the same outcome measure (83), but we were not able to conduct the analyses because of shortage of data. The field would benefit from RCTs using the same outcome measures to include the data needed to conduct a meta-analysis and to have more consensus as to what constitutes psychological distress (14, 20, 82, 83).

We had chosen to use the cut-off point of greater or lesser than 8 weeks as it had been suggested previously in the dementia/Alzheimer's literature but also because it had been suggested that six sessions were considered a therapeutic minimum (13). Therefore, the results suggesting that structure (manual) and duration (at least eight sessions) may be important components of an intervention are in line with previous results (8, 13, 16, 17, 83).

Lastly, we found that the individual and group delivery formats showed an effect when compared to waitlist, treatment as usual, or active control. These results have also been found previously (8, 13). We posit that for some, the group delivery format offers a unique opportunity for social connection and support, and that for others it may not be either possible or suitable to meet in a group. The delivery format (individual or group) may be important when thinking of the intention of the intervention (i.e., provide information about an illness, teach communication or emotion regulation skills, respite care, etc.).

Knight et al. (82) suggested that knowledge of an illness could not be correlated with caregiver distress. We therefore posit that the intervention delivery format may not be as important as the program theory behind the intervention. Interventions developed to provide information to informal caregivers about an illness may not necessarily decrease either symptoms of depression, anxiety, or stress, whereas interventions that are developed to train skills that allow for informal caregiver to address, accept, and regulate difficult emotions experienced because of a loved one's mental illness may decrease symptoms of caregiver depression, anxiety, and stress.

To this point, a recent RCT showed that an 8 week manualized group compassion-based intervention given to informal caregivers of people with all types of mental illness (including dementia, schizophrenia, bipolar, depression, autism, etc.) decreased symptoms of depression, anxiety, and stress and increased overall wellbeing with results lasting at 6 month follow-up (10). The program theory behind manualized compassion-based training programs is to increase a person's ability to deal with and accept difficult emotions (suffering) and feel a motivation and/or wish to relieve the suffering. The results of the RCT suggest that the program theory of the intervention (i.e., practicing compassion for ones own and others suffering thereby training the skills necessary to be with difficult emotions), the fact that the intervention is manualized, at least 8 weeks long, and includes psychoeducation along with active participation (psychosocial) are all effective components in decreasing informal caregiver psychological distress (10). In addition, mediators driving the effect of the intervention were investigated, and the results showed that both self-compassion and mindfulness drove the effect, thereby showing that the intended skills being taught and practiced targeted the intended outcome (i.e., decreasing symptoms of depression, anxiety, and stress) (85). The findings from this research show the importance of having a program theory for an intervention and investigating mediators of change to understand whether the components included in an intervention are the components driving the desired effect. This is in line with the recommendations of the National Institute of Health and the Medical Research Council highlighting the importance of understanding why and how an intervention works (86, 87).

Based on the results found here, we find it appropriate to provide informal caregivers with interventions that aim to reduce psychological distress. However, our review can only provide information regarding the effect of informal caregiver interventions on psychological distress in general and not on any specific intervention modality. Our results indicate that interventions that are manualized and at least 8 weeks long may be important components in creating an effect on informal caregiver psychological distress. Furthermore, our results showed that interventions for informal caregivers of people with dementia/Alzheimer's disease had a small effect, and that interventions for informal caregivers of people with a severe mental illness showed a moderate effect. It may be that age moderates the effect and should be investigated in the future. It may also reflect that the burden due to a severe mental illness is more severe and leaves more room for improvement. We should use caution when interpreting the results because of the substantial heterogeneity, small sample size, and low quality of the included RCTs.

### Implications

The number of informal caregivers is on the rise, and it is paramount that resources and efforts are put into place to prevent psychological distress in caregivers by conducting systematic intervention research on the continuing effectiveness of interventions and implementing the interventions in society. Intervention research would benefit from the use of similar outcome measures (8, 17). Clear descriptions as to the structure and content of the intervention and reporting of means and standard deviations of primary and secondary outcomes may aid in the investigation of which intervention modality is most effective in decreasing caregiver psychological distress. Also, it may be helpful to investigate if duration, manual, and outcome are important for caregiver interventions regardless of mental illness or whether there is a difference depending on the type of mental illness. Lastly, we must understand the mediators that bring about the change in decreasing caregiver psychological distress. A program theory that describes key components and mechanisms may improve caregiver interventions and evidence.

### Strengths and limitations

First, the main strength of this systematic review and meta-analysis is that we provided an overview of the intervention literature regardless of care-receiver illness or intervention modality. In conducting a subgroup analysis of several different categories (i.e., group and individual formats, severe mental illness and dementia/Alzheimer's, longer and shorter durations, and manualized and non-manualized interventions), we have managed to provide a good overview of the literature from its inception to 2019. We have addressed the issue of methodological rigor, by inlcuding only RTCs. It is also a strength that we calculated the SMD from within group changes. In RCTs with small sample size, there is a greater probability of between-group differences in baseline values than in large RCTs. This may confound the effect estimates when only comparing the follow-up data. Third, this study was preregistered with a protocol in PROSPERO, and we followed the PRISMA guidelines. Lastly, we had two independent reviewers for assessing the quality of the RCTs and extracting quantitative data for the meta-analysis.

This review also had several limitations. First, it is limited by the quality of the included RCTs. The majority of the included RCTs had small sample sizes limiting the generalizability of the treatment effect. Second, a substantial statistical heterogeneity (*I*^2^ = 50–90%) was found for the main meta-analysis with interventions being compared with waitlist, treatment as usual, or active control. The majority of the subgroup analyses showed substantial heterogeneity, and interpretation of the results must be taken with care, as the chi-squared test has low power in studies with a low sample size, and a small number of studies are included (70). We conducted sensitivity analyses to understand what created the substantial heterogeneity. For some of the sensitivity analyses, the substantial heterogeneity came from poor-quality RCTs, and for others, it was simply pooling too many RCTs with too many different intervention modalities, delivery methods, durations, and structures. One could even argue that it is futile to conduct a meta-analysis when there is high heterogeneity among the included RCTs (20).

Third, per the PROSPERO protocol, we wanted to conduct subgroup analyses on the intervention modality to understand which intervention modality showed an effect on decreasing informal caregiver distress. Unfortunately, we had to conclude that because of the different and inconsistent component descriptions, we were not able to conduct subgroup analyses on intervention modality, which is a common problem in the literature (20, 83). Fourth, the RCTs used an array of different self-report questionnaires to measure psychological distress, making it difficult to know whether the interventions were addressing the same construct, which is also a common problem in the intervention literature (20, 21). Fifth, it is often quite difficult to assess the risk of bias, as authors often do not specifically report the different processes (e.g., randomization or allocation) in full, which is also a common issue in the field (83). Sixth, while some of the RCTs reported a statistically significant effect of their outcome measure, we were not able to replicate the results in our analysis. We suggest that the discrepancy may be due to the use of different statistical models. Lastly, it was beyond the scope of this meta-analysis to make between-group comparisons.

## Conclusion

The evidence supports that several intervention modalities improve the mental health of caregivers. Manualized interventions, ≥8 weeks, with active participation are most effective. Future RCTs should improve methodology and investigate which intervention modality is most effective for what kind of caregiver and clearly specify what the included intervention components are, use longer follow-up times, and conduct mediational analyses to better understand what mediators create the effect of an intervention. This systematic review and meta-analysis aids in highlighting how great this need truly is.

## Data availability statement

The original contributions presented in the study are included in the article/Supplementary material, further inquiries can be directed to the corresponding author.

## Author contributions

NH, LF, and LJ: overall idea of the article, write-up of article, and data analysis. NH and KP: risk of bias assessment. NH and LB: data extraction. All authors contributed to the article and approved the submitted version.

## Conflict of interest

The authors declare that the research was conducted in the absence of any commercial or financial relationships that could be construed as a potential conflict of interest.

## Publisher's note

All claims expressed in this article are solely those of the authors and do not necessarily represent those of their affiliated organizations, or those of the publisher, the editors and the reviewers. Any product that may be evaluated in this article, or claim that may be made by its manufacturer, is not guaranteed or endorsed by the publisher.
