# Supplementary material for: The effect of mental health interventions on psychological distress for informal caregivers of people with mental illness: A systematic review and meta-analysis
Source: Front Psychiatry. 2022 Oct 6;13:949066. doi: 10.3389/fpsyt.2022.949066 (PMC9583525; doi:10.3389/fpsyt.2022.949066)

# Supplemental Materials

eFigure 1. Sensitivity analysis for meta-analysis and all subgroups

Meta-analysis: all studies


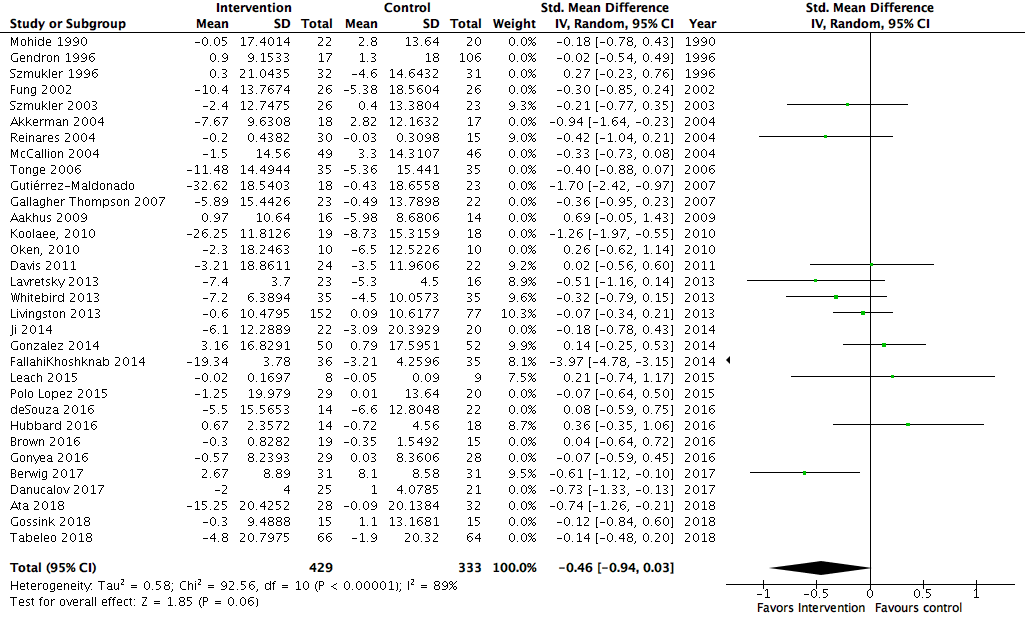


Sensitivity analysis: Dementia/Alzheimer’s disease


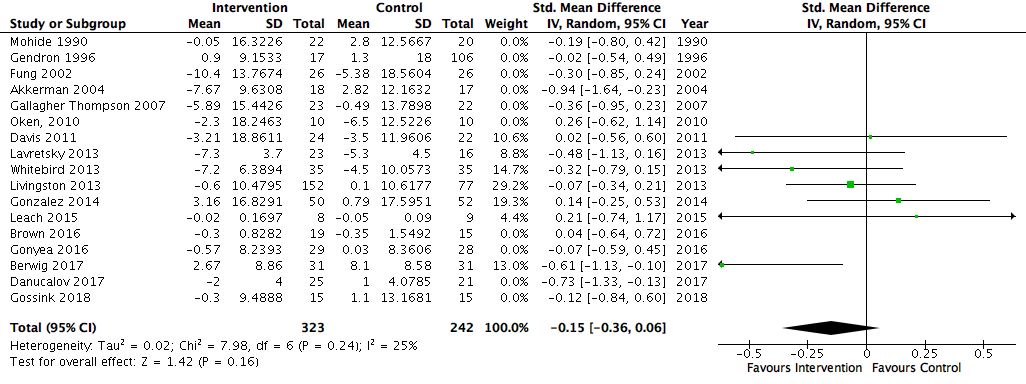


Sensitivity analysis: Severe mental illness


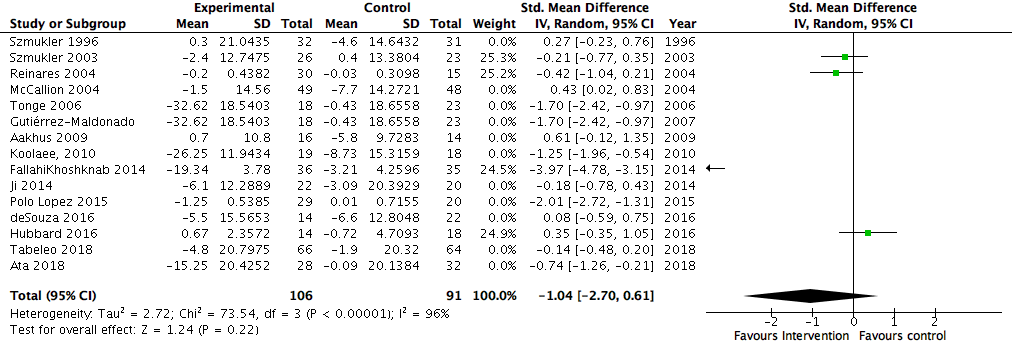


Sensitivity analysis: Individual delivery format


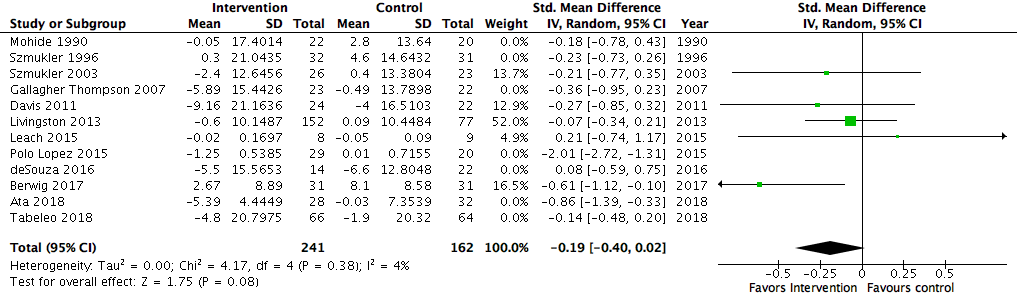


Sensitivity analysis: Group delivery format


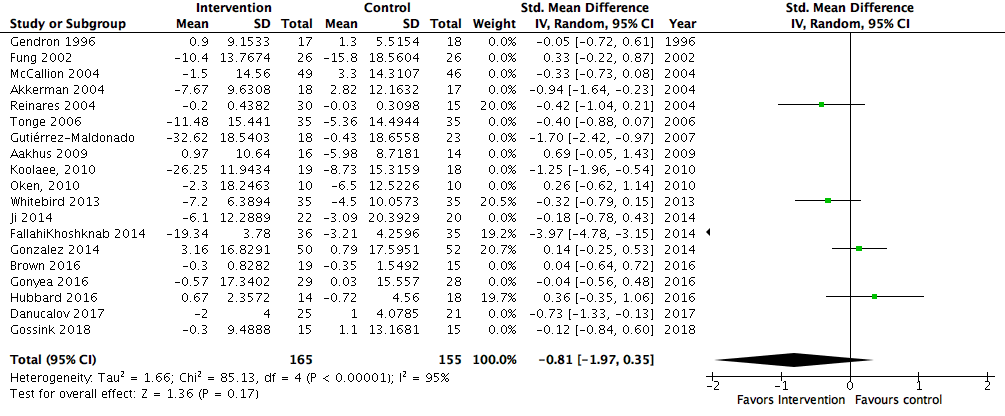


Sensitivity analysis: Manualized, 8 weeks, group/individual


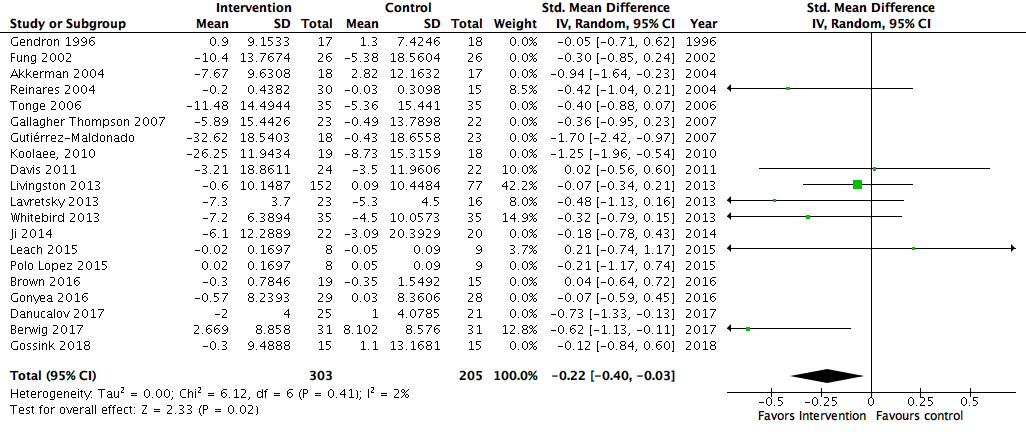


Sensitivity analysis: Non-manualized, less than 8 weeks, group/individual


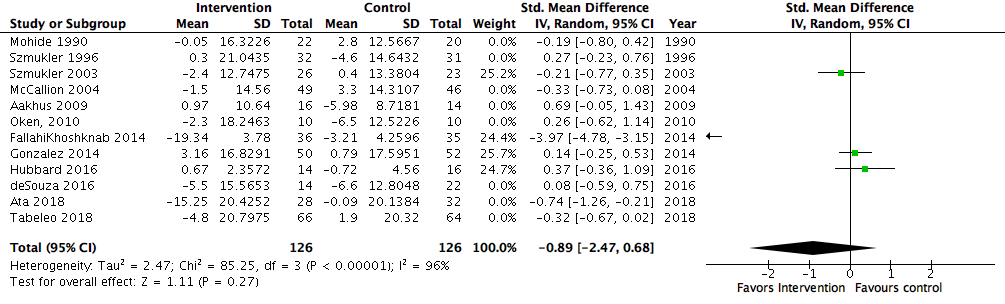


Sensitivity analysis: Active control


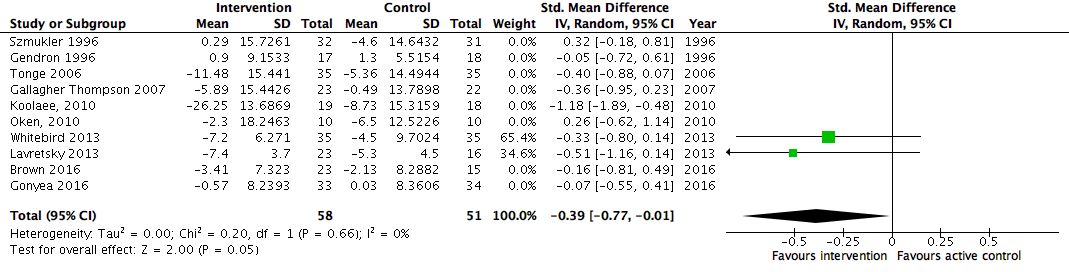

Supplement: Supplementary file 1 [file Data_Sheet_1.docx]
